# High-Throughput Sequencing Reveals Further Diversity of *Little Cherry Virus 1* with Implications for Diagnostics

**DOI:** 10.3390/v10070385

**Published:** 2018-07-21

**Authors:** Asimina Katsiani, Varvara I. Maliogka, Nikolaos Katis, Laurence Svanella-Dumas, Antonio Olmos, Ana B. Ruiz-García, Armelle Marais, Chantal Faure, Sébastien Theil, Leonidas Lotos, Thierry Candresse

**Affiliations:** 1 Laboratory of Plant Pathology, School of Agriculture, Faculty of Agriculture, Forestry and Natural Environment, Aristotle University of Thessaloniki, 54124 Thessaloniki, Greece; akatsian@uark.edu (A.K.); katis@agro.auth.gr (N.K.); llotos@agro.auth.gr (L.L.); 2 UMR 1332 Biologie du Fruit et Pathologie, INRA, University of Bordeaux, CS20032, Villenave d’Ornon CEDEX, F-33882 Bordeaux, France; laurence.svanella-dumas@inra.fr (L.S.-D.); armelle.marais-colombel@inra.fr (A.M.); chantal.faure@inra.fr (C.F.); sebastien.theil@inra.fr (S.T.); thierry.candresse@inra.fr (T.C.); 3 Centro de Protección Vegetal y Biotecnología, Instituto Valenciano de Investigaciones Agrarias (IVIA), Ctra. Moncada-Naquera km 4.5, Moncada, 46113 Valencia, Spain; aolmos@ivia.es (A.O.); ana.belen.ruiz@uv.es (A.B.R.-G.)

**Keywords:** LChV1, *Closteroviridae*, intra-host variability, high-throughput sequencing, diagnostics

## Abstract

*Little cherry virus 1* (LChV1, *Velarivirus*, *Closteroviridae*) is a widespread pathogen of sweet or sour cherry and other *Prunus* species, which exhibits high genetic diversity and lacks a putative efficient transmission vector. Thus far, four distinct phylogenetic clusters of LChV1 have been described, including isolates from different *Prunus* species. The recent application of high throughput sequencing (HTS) technologies in fruit tree virology has facilitated the acquisition of new viral genomes and the study of virus diversity. In the present work, several new LChV1 isolates from different countries were fully sequenced using different HTS approaches. Our results reveal the presence of further genetic diversity within the LChV1 species. Interestingly, mixed infections of the same sweet cherry tree with different LChV1 variants were identified for the first time. Taken together, the high intra-host and intra-species diversities of LChV1 might affect its pathogenicity and have clear implications for its accurate diagnostics.

## 1. Introduction

*Little cherry virus 1* (LChV1), a member of the genus *Velarivirus* (family *Closteroviridae*), is a graft-transmissible pathogen and its host range includes mainly sweet or sour cherry and other *Prunus* species [1]. Besides the typical reddening frequently observed in cherry leaves, some LChV1 isolates have been associated with various plant disorders [2,3]. LChV1 has a long, positive-sense, single-stranded RNA genome, which encodes eight open reading frames (ORFs) [4]. ORF 1a and ORF 1b, with ORF 1b expressed by a frameshift of ORF 1a, encode a large protein with papain-like proteinase (P-PRO), methyltransferase (MET) and Helicase (HEL) domains and a protein containing an RNA-dependent RNA polymerase (RdRp) conserved domain, respectively. ORF 2 encodes a small hydrophobic protein (4 kDa) which partially overlaps with the ORF 3 that encodes a 70 kDa heat-shock protein 70 homolog (HSP70h). ORF 4 encodes a polypeptide of 61 kDa and partially overlaps with ORF 3, ORF 5 and ORF 6 encode the coat protein (CP) and the CP minor (CPm), respectively, whereas ORF 7 and ORF 8 encode polypeptides of respectively 21 and 27 kDa, with currently unknown functions.

LChV1 exhibits high genetic diversity as revealed from partial or full genome sequencing of various isolates. So far, the complete genome of several LChV1 isolates has been determined [2,3,4,5,6,7]. The UW2 and ITMAR isolates are highly similar, whereas isolate V2356 differs significantly from them and represents the first fully sequenced one of a second phylogenetic group which also includes isolates from the US [2,3]. A third group is formed with the isolates Jerte, Ponferrada, Taian and YD [5,6,7]. Phylogenetic analyses using partial sequences of the RdRp, the HSP70h and the CP have classified LChV1 isolates into 4 clusters, including the three above mentioned ones [8]. Even though the intragroup diversity appears to be relatively low (3.3% to 7.4% in nts), the genetic distances between the different phylogenetic groups are higher (15% to 39% in nts) [3,5,6,7,8].

High throughput sequencing (HTS) has been used in plant virology since 2009 [9,10]. HTS provides highly efficient, rapid and cost-effective sequencing of the genomes of plant viruses and viroids [11]. Different approaches, such as sequencing of total RNA, double-stranded RNA (dsRNA) or small RNAs (sRNAs) have been developed. HTS was used in a number of studies in plant virology including, but not limited to, discovery of novel viruses and viroids as well as analysis of genome diversity and evolution, and study of pathogen epidemiology and population structure [12,13,14]. Another area where HTS has proven very valuable is in the detection of divergent variants of known viruses that escape existing detection procedures, particularly PCR or RT-PCR assays [15]. Because of its unbiased nature, the data obtained by HTS may provide a better knowledge of the polyvalence or specificity of existing assays and, if needed, facilitate the design of new detection primers of broader specificity, thus contributing to the improvement of classical detection methods.

In the present study, seven novel LChV1 isolates were fully sequenced using HTS technologies. One of the isolates (G15 3) was recently partially characterized and shown to exhibit the highest genetic divergence from all so far known isolates [8]. The sequences of the other LChV1 isolates described herein were acquired during HTS analysis of several sweet or flowering cherry samples from three different countries. Our results highlight the presence of further genetic diversity in the populations of LChV1, with clear implications for the diagnosis of this viral agent of quarantine status or included in cherry certification in a number of countries.

## 2. Materials and Methods 

### 2.1. Full Genome Sequencing of LChV-1 Isolates Using HTS Approaches

Three sweet cherry and one flowering cherry samples from Greece (G15 3, C118), Spain (P8) and Japan (Kyoto-2) were analyzed using different HTS approaches (Table 1). The G15 3 isolate (LN794218) was obtained from a sweet cherry tree (cv. Tragana Edessis) which did not show any virus-like symptoms. The sample was collected in 2009 from a Greek stone fruit orchard (Aridaia Pella) and a part of the isolate’s sequence was previously determined, showing high divergence from other known LChV1 isolates [8]. The Kyoto-2 isolate (MG934545) was obtained from a flowering cherry (*Prunus serrulata*) with conspicuous yellowish oak-leaf pattern symptoms collected in late spring 2015 in the Kyoto prefecture of Japan. The C118-Iso1 (MH364114), C118-Iso13 (MH364115) and C118-Iso15 (MH364116) isolates were obtained from C118, a sweet cherry tree (cv. Larian) showing no obvious symptoms, collected in summer 2014 from a Greek nursery in Rizari (Pella) and maintained in the premises of the Laboratory of Plant Pathology until sequenced in 2017. The P8-23 (MH300060) and P8-42 (MH300061) isolates were obtained from P8, a sweet cherry tree (cv. Planera) showing reddening of the leaves collected in early summer 2017 in Alicante, Spain.

The samples were subjected to total RNA, small RNAs (sRNAs) or double-stranded RNA (dsRNA) extraction before their HTS analysis (Table 1). More specifically sRNAs were isolated from leaf midribs of the G15 3 infected sweet cherry tree using the mirPremier microRNA Isolation Kit (Sigma-Aldrich, St. Louis, MO, USA). Double-stranded RNAs were extracted from *P. serrulata* symptomatic leaves as previously reported (3) and total RNA was extracted from leaf and petiole tissue of the C118 and P8 samples using the Plant/Fungi total RNA purification kit (NorgenBiotek Corporation, Thorold, ON, Canada) according to the manufacturer’s protocol.

cDNA libraries and HTS of G15 3 were performed by LifeSequencing S.L. (Paterna, Spain) on an Ion Torrent platform using the Ion chip 318. Complementary DNA obtained from the purified dsRNAs of Kyoto2 was sequenced in multiplex using the Illumina Miseq platform. Finally, the samples C118 and P8 were subjected to rRNA depletion, library construction and high throughput sequencing (150 bp paired-end reads, total output 20 million) in a NextSeq 500 platform (Illumina, San Diego, CA, USA) at Life Sequencing S.L. (Paterna, Spain). In all cases the obtained reads were de novo assembled using CLC Genomics Workbench v.10.1.1 (Qiagen Bioinformatics, Hilden, Germany). For the total RNA reads prior to de novo assembly the host genome was removed using the same software. In addition, in the case of G15 3 all sRNAs used for the construction of the contigs, were mapped on the V2356, ITMAR and UW2 isolate sequences. The de novo contigs produced were blasted (BLASTn/x) against local and online virus, viroids and nt/nr databases.

In order to confirm the G15 3 and Kyoto-2 sequences obtained from HTS data and to fill sequence gaps, primer pairs were designed from contig sequences (Table S1) and used in RT-PCR assays. More specifically, in the case of G15 3, the cDNA was synthesized by adding 5 μL of total RNA in the RT mixture [250 mΜ Tris-HCl (pH 8.3), 375 mM KCl, 15 mM MgCl2, 0.5 mM of each (dNTP), 0.4 μΜ of random hexameres 5′ d(N6) 3′ [N = A,C,G,T] and 60 units of Superscript^®^ II (Invitrogen, Gröningen, The Netherlands)] in a total volume of 20 μL. The reaction was performed under the following profile: 15′ in 18 °C, 40′ in 37 °C, 15′ in 42 °C and 5′ in 95 °C. Five μL of the cDNA were added in the PCR mixture containing 10 mM Tris–HCl (pH 8.8), 50 mM KCl, 1.5 mM MgCl_2_, 0.1% Triton X-100, 5% DMSO, 0.2 mM of each dNTP and 0.5 units Dynazyme™ II DNA Polymerase and 0.4 μΜ from each primer (Table S1). The cycling profile consisted of a step at 95 °C for 5 min, and 40 cycles segmented in 30 s at 95 °C, 30 s at 55 °C and 60 s at 72 °C, followed by one final extension step at 72 °C, for 5 min.

In case of Kyoto-2, complementary DNAs were synthesized from 3 µL of purified dsRNAs using N6 and dT18 primers and the Superscript II Reverse Transcriptase (Invitrogen, Gröningen, The Netherlands), according to the manufacturer’s recommendations. In a second step, the PCR amplification was carried out using 2 µL of cDNA in a 50 µL reaction volume, 0.2 µM of each specific primer and 1 µL of 50× Advantage 2 Polymerase mix (Clontech, Mountain View, CA, USA) according to the manufacter’s recommendations. The 5′ and 3′ genome ends of Kyoto-2 isolate were determined using specific primers (Table S1) designed from terminal contig sequences and Rapid Amplification of cDNA Ends on purified dsRNAs (RACE, Takara Bio Europe/Clontech, Saint-Germain-en-Laye, France). All amplifications products were sequenced by Genewiz (Paris, France).

### 2.2. Phylogenetic and Sequence Analysis of LChV1 Isolates

For the analysis of the sequences determined in this study, all full-length genome sequences available from the EMBL-EBI and GenBank databases, as well as partial sequences included in a recent phylogenetic analysis [8] were used. ORFs were determined with “ORF finder” (http://www.bioinformatics.org/sms2/orf_find.html) (Department of Biological Sciences, University of Alberta, Edmonton, Canada) and the identity scores in aminoacids and nucleotides with the GeneDoc software version 2.7.0 (Copyright (C) 2006, Karl Nicholas) [16]. Multiple sequence alignments were performed with Muscle (MUltiple Sequence Comparison by Log-Expectation) [17]. Phylogenetic trees were constructed using either full genomes or the partial RdRp, HSP70h and CP sequences with the Maximum Likelihood (ML) algorithm implemented in MEGA v. 7.1 (Molecular Evolutionary Genetics Analysis) [18], applying GTR+I+G, T92+G+I, T92+I and T92+G nucleotide substitution models, respectively, and 500 bootstrap replicates.

### 2.3. Recombination Analysis

The detection of recombination events was performed with RDP v.4.95 (Recombination Detection Program) [19] under default conditions, using an alignment of complete LChV1 genome sequences constructed with MAFFT v7 (Multiple Alignment using Fast Fourier Transform) [20].

### 2.4. In Silico Evaluation of Published Primers for the Detection of LChV1

The sequences of all primers reported to date for the detection of the virus were compared in silico with LChV1 complete genome sequences in order to determine the primer pair exhibiting the broadest detection range (Figure 4) [2,3,7,8,21,22,23,24,25].

## 3. Results

### 3.1. HTS Analyses and Genome Assembly of LChV1 Isolates

HTS of the G15 3 sample using the Ion Torrent platform provided a total of 3,971,532 sequence reads among which 32,358 or 0.81% were sRNAs of 21–24 nt related to LChV1. Blast analysis of the contigs constructed by the de novo assembly of the reads indicated the presence of LChV1 along with other *Prunus* infecting viruses (Table 1).

**Figure 1:**
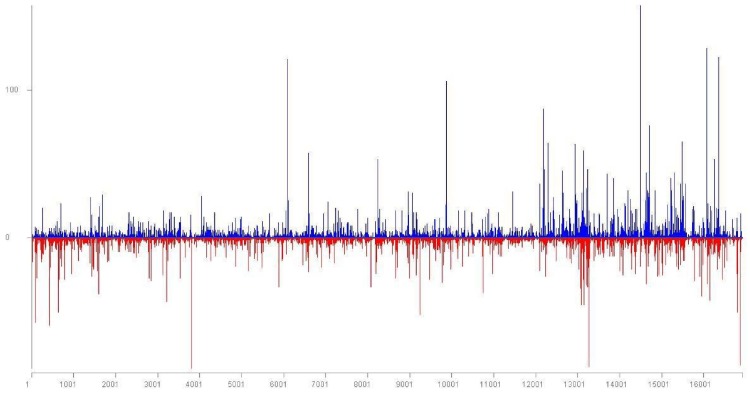
Location and frequency of *Little cherry virus 1* (LChV1) small RNA reads on the LChV1 genomic RNA. Positive-sense reads are shown as blue bars; negative-sense reads are shown as red bars below the x-axis.

A total of 71 LChV1 contigs (length higher than 50 nts) were identified by Blast analysis. In parallel, all sRNAs integrated in these contigs were mapped with the Geneious (Biomatters Ltd., Auckland, New Zealand) software on the complete genomes of the ITMAR, V2356 and UW2 isolates. A full coverage of the LChV1 genome was thus obtained using these sRNA sequences obtained by HTS. A relatively higher number of LChV1 sRNAs were allocated (+ and − polarity) in the 3′ region of the gRNA and in selected spots of the genome (Figure 1). The G15 3 genome sequence was confirmed with Sanger sequencing using specific primers (Table S1).

Following the de novo assembly of the LChV1 Kyoto-2 reads using CLC Genomics Workbench 9.0 (Qiagen Bioinformatics, Hilden, Germany), BlastN and BlastX analyses (cut-off value of 10^−3^) revealed the presence of contigs belonging to several well-known fruit tree viruses, including LChV1 (Table 1). Since the Blast analyses indicated that the LChV1 Kyoto-2 isolate was highly divergent from isolates present in GenBank, a specific effort was made to assemble the 15 LChV1 contigs identified (representing 1062 reads or 0.98% of total reads for this sample) in a scaffold covering most of the virus genome. Specific primers designed from the sequence of the contigs (Table S1) were used to fill the 10 gaps in the scaffold and sequence the missing genome ends. 

De novo assembly with CLC Genomics Workbench (Qiagen Bioinformatics, Hilden, Germany) of 56,727,134 total RNA reads from the Greek sweet cherry sample C118 generated 3 almost full-genome sequences for LChV1 (0.07% or 37,874 reads for C118-Iso1, 0.05% or 27,114 reads for C118-Iso13 and 0.04% or 25,494 reads for C118-Iso 15) (16.662, 16.812 and 16.878 nt), along with other contigs belonging to several fruit tree viruses (Table 1). Blast analyses of these LChV1 contigs showed high similarities with three distinct LChV1 isolates, indicating a mixed infection with different viral genotypes.

Likewise, de novo assembly with CLC Genomics Workbench (Qiagen Bioinformatics, Hilden, Germany) of 54,261,654 total RNA reads from the Spanish sweet cherry sample P8 generated 2 full-genomes for LChV1 (7.2% or 388,269 reads for P8-23 and 3.0% or 161,431 reads for P8-42) (16.938 and 16.963 nt), along with other contigs belonging to several fruit tree viruses (Table 1). Blast analyses of these contigs showed high similarities with two different LChV1 isolates, indicating a mixed infection with different viral genotypes.

### 3.2. Genomic Organization and Sequence Similarities of the New LChV1 Isolates

LChV1 G15 3 (16.880 nt) and Kyoto-2 (16.927 nt) genomic sequences were deposited in the GenBank under the accession numbers LN794218 and MG934545, respectively. Based on Blast analysis these two isolates represent divergent genotypes of LChV1 and therefore their genome organization was further analyzed in detail. The variants obtained from the C118 (MH364114, MH364115, MH364116) and P8 (MH300060, MH300061) sources had the typical genome organization of LChV1 and showed high identity rates (90–98% in nts) with already sequenced variants present in GenBank (Table S2).

**Figure 2:**
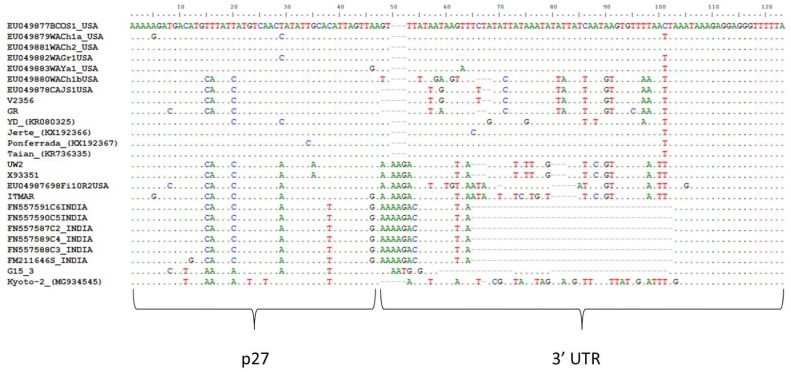
Multiple sequence alignment representing the beginning of the 3′ Untranslated Region (UTR). All available in GenBank LChV1 isolates with deposited sequences from this genomic region, were used.

The G15 3 and Kyoto-2 genomes showed overall nucleotide identity percentages that ranged between 72–73% and 75–77%, respectively, with the other available LChV1 full genomes, whereas they shared 73% nt identity (Table 2). The amino acid identity percentages ranged between 65–92% and 70–94% for G15 3 and Kyoto-2, respectively, with the other available full genomes and 68–92% between them. In the 5′ Untranslated Region (UTR) of the G15 3 and Kyoto-2 genomes a limited number of indel polymorphisms were observed when compared to reference sequences. In fact, this small genomic region was found to be highly conserved between LChV1 isolates. ORF1a, which encodes a large protein with P-PRO, MET and HEL conserved domains, shares 70–72% (G15 3) and 71–76% (Kyoto-2) identities in nt with the already characterized LChV1 isolates (Table 2). ORF1b, which encodes the viral RdRp shares higher nucleotide similarities with other isolates, 80–81% and 79–83% for G15 3 and Kyoto-2, respectively (Table 2). The P4 proteins of the G15 3 and Kyoto-2 isolates are 5 amino acids (aa) shorter than those of the UW2 and ITMAR isolates, as a point mutation of a thymine (T) to adenine (A) results in the creation of a premature stop codon. The genomic sequence encoding this protein shares respectively 70–81% (G15 3) and 78–81% (Kyoto-2) nt identity with other isolates. Both for G153 and Kyoto-2, a 1 nt insertion within ORF3 (encoding the HSP70 homolog) results in a frameshift and the production of a 67 aminoacids shorter protein when compared to the UW2 and ITMAR isolates. In ORF4 (P61) and ORF5 (CP), the level of nucleotide divergences with other LChV1 isolates remains high, reaching only for the P61 ORF 72–74% nt identity (G15 3) to 73–76% (Kyoto-2) and for the CP gene 70–72%nt identity (G15 3) to 71–78% (Kyoto-2) (Table 2). In both isolates a 1 nt deletion towards the 3′ end of ORF6 (CPm) leads again to premature termination and, in this case, to a protein 2 aminoacids shorter as compared to the UW2 and ITMAR isolates. Again, high divergence levels are observed in this ORF (68–70% and 70–73% nt identity with other LChV1 isolates for the G15 3 and Kyoto-2 isolates, respectively) (Table 2). The indel polymorphisms and the mutations mentioned above were also observed in the rest of the isolates sequenced in the present study, as well as in all recently characterized LChV1 isolates [3,5,6,7,8]. An ORF finder analysis placed the beginning of ORF7 (P21) at position 15.285 of the genome when the alignment of all available full-length genomes was used (position 15.254 on the G15 3 genome). However, ORF7 for ITMAR and UW2 starts only at position 15.436, because a C insertion at position 15.402 results in a frameshift in the 5′ region of the ORF, so that initiation is only possible on the downstream methionine, resulting in a 50aa shorter protein. Similarly, ORF8 (P27) starts at position 16.018 of the alignment of full genomes, whereas for the YD isolate the insertion of an A at position 16.044 forces initiation of the ORF at a downstream methionine at position 16.076, resulting in a 19aa shorter protein. For both G15 3 and Kyoto-2, the level of nucleotide divergence with other isolates was higher in ORF8 (76–78% for G15 3 and 77–85% for Kyoto-2, respectively) than for ORF7 (71–75% for G15 3 and 74–81% for Kyoto-2, respectively) (Table 2). Finally, in the first 50 nucleotides of the 3′ UTR (~200 nt long) a high variability is observed between all LChV1 isolates (Figure 2). In addition, indel polymorphisms were observed in several positions of the 3′ UTR. In G15 3, deletions were observed in a significant number of positions within the 3′ UTR, including 13 nt and 23 nt long deletions. Similar deletions were only observed in LChV1 isolates from India (Figure 2). The divergent region is followed by a highly conserved region among all isolates. 

### 3.3. Intra-Host Genetic Diversity of LChV1

A mixed infection with different LChV1 genotypes was identified in samples C118 and P8 from Greece and Spain, respectively. The C118-Iso1 sequence shares 99% identity with the G15 3 described in the present study. The C118-Iso13 sequence shares 98% identity with the V2356 isolate while the C118-Iso15 sequence shares 94% and 92% identity, respectively, with isolates UW2 and ITMAR (Table S2). The P8-23 sequence shares 97% identity with the Ponferrada, Jerte and Taian isolates. The P8-42 shares 90% identity with the V2356 isolate (Table S2). The co-infection of the sweet cherry trees with different LChV1 isolates was confirmed by RT-PCR amplification and RFLP analysis of the amplified CP gene (Supplementary Materials).

### 3.4. Recombination Analysis

**Figure 3:**
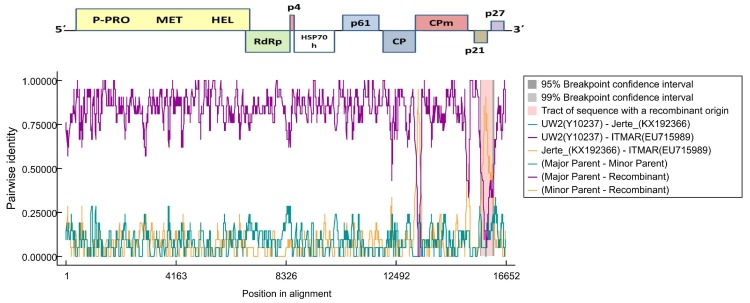
Recombination events predicted by RDP v.4.95 (Recombination Detection Program) in the 3′ end of the ITMAR isolate of LChV1. Lines in the graph give the pairwise identity percentage in the respective alignment position. The recombinant part is indicated by a frame and the breakpoints by darker background. LChV’s genome is given above the graph.

A recombination analysis performed with RDP4 on an alignment of all available full-length LChV1 genomic sequences revealed the presence of two recombination events in the 3′ part of the ITMAR genome and UW2 and Jerte are presumed to be the parental isolates (Figure 3). These events were detected by six out of the seven algorithms used (RDP, GENECONV, BootScan, MaxChi, Chimaera, 3Seq) [26,27,28,29,30]. No clear evidence of recombination was observed for the LChV1 sequences reported in the present study.

### 3.5. Phylogenetic Analysis

**Figure 4:**
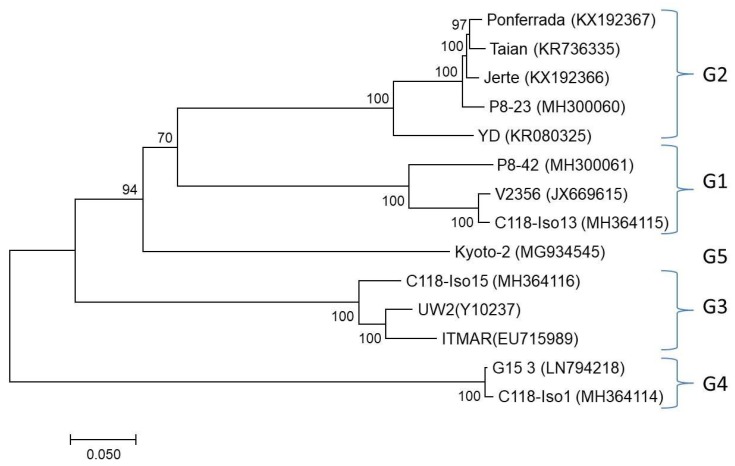
Maximum likelihood phylogenetic tree inferred from the complete nucleotide sequences of LChV1 isolates. All isolates are reported with their names followed by their accession numbers. The tree is midpoint-rooted. The numbers above or below each branch are the nonparametric bootstrap (NPB) values given as percentages of 500 replicates. Only NPB values with *p* > 70 are shown.

A full-length genome phylogenetic analysis clustered all LChV1 isolates (available from GenBank and the reported here) in 5 distinct clusters (Figure 4). In order to compare these results with the grouping proposed in a previous study [8] phylogenetic trees were also constructed using partial RdRp (~700 nt), HSP70h (500 nt) and CP (550 nt) sequences including the partial sequences used in that 2015 study. Comparison of the trees showed that all four previously identified LChV1 clusters were comparably detected in the new analysis but that a fifth cluster was identified formed by the single Kyoto-2 isolate (Figure 4 and Figure S1). 

### 3.6. Detection Range of LChV1 Specific Primers

**Figure 5:**
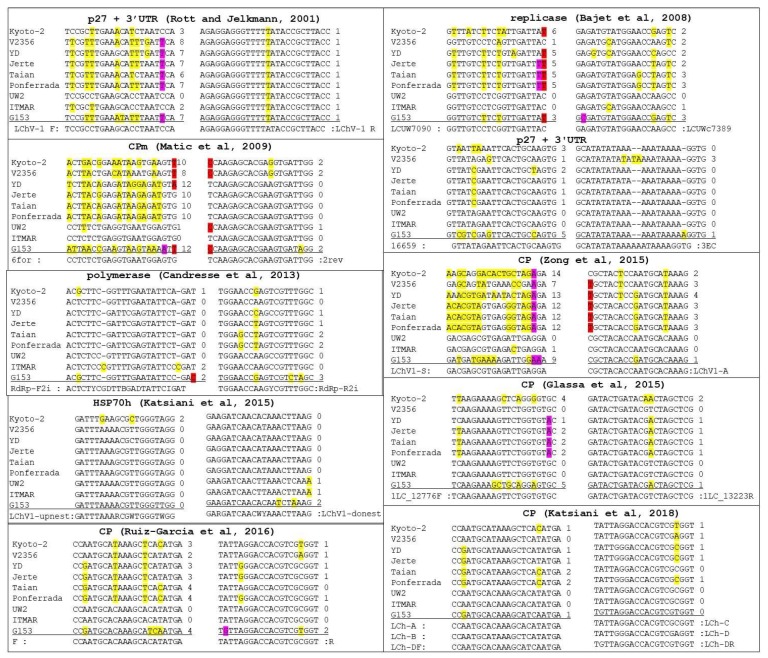
Multiple sequence alignments of the primer binding regions for the detection of LChV1. The position and number of mismatches with the full genome LChV1 isolates representing all phylogenetic groups are indicated. In red, violet and yellow color are presented the mismatches of the primer affecting the first 3′-most nucleotide, the second and third 3′-most nucleotide as well as all other nucleotides, respectively.

To determine the most suitable primer pair for the accurate detection of all isolates of LChV1, detection primer pairs reported in the literature were retrieved and compared with all available full-length genomic sequences (Figure 5). In most cases, these detection primers showed either multiple mismatches with some isolates (up to 10–12 mismatches for the 6 for primer of Matic et al. [2]) or mismatches affecting the two 3′-most nucleotides of the primer (in particular the LCUW7090 primer of Bajet et al. [22]). Such mismatches are highly likely to either preclude amplification of some isolates or severely affect the sensitivity of detection. Taking into account a conservation rule of less than 3 mismatches for a primer with any particular isolate and of no mismatch affecting the 3′ nucleotide of a primer, only two primer pairs are expected to be able to show very broad amplification of all LChV1 isolates (Figure 5), the LChV1-upnest and LChV1-donest pair developed and used in a nested RT-PCR detection scheme targeting the HSP70h [8] and the complex primers mix (LCh-A, LCh-B, LCh-C, LCh-D, LCh-DF, LCh-DR) used in a real-time RT-PCR detection scheme targeting the CP [25].

## 4. Discussion

In this study the complete genome sequences of several LChV1 isolates from three countries was determined. For this purpose, different HTS approaches, which offer fast and accurate determination of genomic sequences, were used [3,31]. Based on the obtained HTS data we were able to obtain the full genomes for two novel and divergent LChV1 isolates, G15 3 and Kyoto-2, as well as the genomes of variants sharing high similarities with previously described ones (C118-Iso1, -Iso13, -Iso15, P8-23, P8-42).

Phylogenetic analysis of the obtained genomic sequences confirmed the existence of high genetic diversity among LChV-1 isolates, which could be clustered in five distinct clades that are not correlated with the geographic origin of the isolates (Figure 4). Interestingly, mixed infections of sweet cherry trees involving different LChV1 genotypes were identified here for the first time. Since no known vector has been found for LChV1, the coexistence of these different virus variants could be attributed to grafting practices involving infected plant material. These mixed infections might have implications on the pathogenicity of the virus and could lead to recombination events as has already been reported for LChV1 [8] and is further described here. 

The demarcation criteria of viral species within the family *Closteroviridae* are based on molecular and biological characteristics as well as on phylogenetic relationships [32]. Due to the high genetic diversity observed for several members of the family, the level of sequence divergence was recently raised from 10 to 25% aa in phylogenetically informative proteins (RdRp, HSP70h, or CP) for species demarcation [32]. Isolate G15 3 is thus close to this species demarcation level in its HSP70h (17–21%), while its CP exceeds it (26–28% with all isolates) (Table 2 and Table S2). However, its divergence with all other LChV1 isolates is much lower in the RdRp (8–10%), confirming that G15 3 belongs to the LChV1 species and represents the first fully characterized isolate of a fourth phylogenetic group, genetically distinct from all others reported so far. Similarly, the Kyoto-2 isolate represents the first fully characterized isolate of a fifth group since its divergence with other isolates in the RdRp was 6–9%, in HSP70h 13–19% and in CP 28% with G15 3 and 21–25% with all other isolates.

Sequence comparisons of all available LChV1 isolates have shown a high conservation in the 5′ UTR and in the 3′-most part of the 3′ UTRs, whereas, a significant number of indel polymorphisms were observed in the 5′-most part of the 3′ UTR of isolate G15 3. Apart from G15 3, deletions in the same positions are also observed in a LChV1 group of isolates from India for which only very partial sequence (409 nt) is available. However, G15 3 was found to be genetically distant from these isolates (nucleotide divergence 28–29% in the sequenced region). Similar large indel polymorphisms have also been reported in the 3′ UTR of some *Grapevine leafroll-associated virus* 2 (GLRaV2) variants [33]. Similar to the situation for GLRaV2, a highly conserved region was identified in LChV1 downstream of the polymorphic region of the 3′ UTR. It is known that the 3′ UTR of positive strand RNA viruses generally contains regulatory sequences essential for the synthesis of the complementary minus strand [33,34]. However, the biological significance of the upstream polymorphisms, if any, remains unknown although it has been speculated that differences in the 3′ UTR might affect the efficiency of the viral genome replication [33].

Within both the G15 3 and Kyoto-2 sequences a small number of shared polymorphisms confirmed by Sanger sequencing were observed affecting several ORFs and leading to either shorter or longer proteins as compared to some other reference isolates. In particular, proteins p4 and CPm are slightly smaller for the Kyoto-2 and G15 3 isolates as compared to the UW2 and ITMAR isolates while their HSP70h is truncated by 67 aminoacids. The same polymorphisms were observed in all other fully characterized LChV1 isolates as well as in some partial sequences [3,5,6,7,8]. It is known that the HSP70h is involved in cell-to-cell movement, assembly of the complexes of the subunits for viral replication and/or synthesis of subgenomic (sg) RNAs, as well as the assembly of the viral particle [35,36,37]. The loss of the C-terminal part of HSP70h in these isolates indicates that this part of the protein is possibly not essential for its function. This finding warrants further studies since it might endow these isolates novel biological properties. Variation in the size of some proteins has also been reported between *Citrus tristeza virus* (CTV) isolates [38].

Proteins p21 and p27, located at the 3′-portion of the genome, were found to be conserved among the LChV1 isolates, whereas no sequence similarity was seen with the similarly sized proteins encoded by the 3′-portions of the genomes of other velariviruses despite extensive BLAST searches). The high variability of these proteins between viruses could reflect specific host adaptation functions such as countering antiviral defenses as found in other closteroviruses [39,40]. Recent preliminary data indicated that p21 is putatively acting as an RNA silencing suppressor [41] however further studies are needed in order to clearly define the role of these two divergent proteins on the infection cycle of LChV1.

The high genetic diversity of LChV-1 and the highly divergent isolates reported here could affect the reliable detection of viral isolates. Indeed, an in silico analysis of the detection primer pairs reported in nine different publications revealed in most cases a significant number of mismatches with at least some LChV1 isolates, to the extent that the amplification of these isolates is likely compromised, limiting the polyvalence of the detection assays employing them (Figure 5). This analysis also showed that the primers used in two detection schemes targeting the HSP70h or the CP [8,25], likely exhibit the highest detection range. 

Although growing evidence suggests that LChV1 isolates could be largely latent in many of their hosts it is still included in many certification and quarantine schemes and several LChV1 isolates have been tentatively associated with specific syndromes in sweet cherry and in other *Prunus* species [2,3,22]. Regarding the isolates analyzed in the present study, no clear conclusions can be drawn concerning their pathogenicity because as is very frequently the case the trees hosting them were co-infected with several viruses including in some cases different LChV1 genotypes. However, the LChV1 C118-iso13 and P8-42 isolates identified here show close phylogenetic relationships with the V2356 isolate which was strongly suggested to cause the Shirofugen stunt disease (SSD) [3], whereas the C118-iso15 variant grouped along with isolate ITMAR which was suggested to be involved in the Kwanzan stunting syndrome [2].

## Figures and Tables

**Table 1:** Description of analyzed samples and viral species identified with high throughput sequencing (HTS).

Sample ID	Plant Species	Country	Symptoms	HTS Template		Viruses Present							
					APCLSV	CVF	PDV	LChV1	LChV2	CVA	PBNSPaV	CNRMV	APLPV
G15 3	*Prunus avium* cv. Tragana-edessis	Greece	no	sRNAs		+	+	+					
Kyoto-2	*Prunus serrulata*	Japan	yellowish oak-leaf pattern symptoms	dsRNA				+	+	+	+	+	+
C118	*Prunus avium* cv.Larian	Greece	no	Total RNA	+			Multiple genotypes		+		+	
P8	*Prunus avium* cv.Planera	Spain	reddening of leaves	Total RNA			+	Multiple genotypes		+			

HTS: High-throughput sequencing, sRNAs: small RNAs, dsRNA: double-stranded RNA, APCLSV: *Apricot pseudo-chlorotic leaf spot virus*, CVF: *Cherry virus F*, PDV: *Prune dwarf virus*, LChV1: *Little cherry virus 1*, LChV2: *Little cherry virus 2*, CVA: *Cherry virus A*, PBNSPaV: *Plum bark necrosis and stem pitting-associated virus*, CNRMV: *Cherry necrotic rusty mottle virus*, APLPV: *American plum line pattern virus*. The viral species identified in the samples are indicated with “+”.

**Table 2:** Nucleotide and aminoacid identity percentages (%) between the G15 3 and Kyoto-2 isolates with all other fully sequenced LChV1 isolates.

**G15 3**	**Identity in nt (aa) with LChV-1 Isolates**							
**Genomic Region**	**ITMAR**	**UW2**	**V2356**	**YD**	**JERTE**	**PONFERRADA**	**TAIAN**	**KYOTO-2**
Full genome	72	72	72	73	73	73	73	73
ORF1a (P-PRO, MET & HEL)	70 (75)	70 (76)	72 (76)	72 (76)	72 (77)	71 (76)	71 (76)	71 (76)
ORF1b (RdRp)	80 (90)	80 (92)	81 (92)	80 (92)	80 (91)	81 (92)	80 (90)	80 (92)
ORF2 (p4)	72 (67)	70 (67)	76 (77)	76 (70)	74 (70)	70 (67)	73 (74)	81 (83)
ORF3 (HSP70h)	75 (79)	76 (81)	74 (81)	76 (83)	76 (83)	76 (83)	76 (83)	76 (81)
ORF4 (p61)	72 (75)	72 (75)	74 (76)	72 (75)	73 (74)	73 (74)	73 (74)	73 (74)
ORF5 (CP)	72 (73)	72 (72)	70 (72)	72 (73)	72 (74)	72 (74)	72 (74)	71 (72)
ORF6 (CPm)	69 (66)	70 (68)	68 (65)	69 (68)	69 (67)	69 (67)	70 (67)	70 (68)
ORF7 (p21)	76 (88)	76 (87)	76 (85)	78 (87)	78 (88)	78 (88)	78 (88)	77 (85)
ORF8 (p27)	71 (69)	73 (74)	74 (74)	73 (71)	73 (75)	73 (75)	72 (75)	75 (71)
**KYOTO-2**	**Identity in nt (aa) with LChV-1 Isolates**							
**Genomic Region**	**ITMAR**	**UW2**	**V2356**	**YD**	**JERTE**	**PONFERRADA**	**TAIAN**	**G15 3**
Full genome	75	75	77	77	77	76	77	73
ORF1a (P-PRO, MT & HEL)	74 (79)	74 (80)	76 (83)	75 (83)	75 (83)	75 (82)	75 (82)	71 (76)
ORF1b (RdRp)	79 (91)	80 (93)	83 (94)	80 (94)	81 (93)	81 (93)	81 (93)	80 (92)
ORF2 (p4)	79 (70)	82 (77)	81 (80)	81 (70)	79 (70)	78 (74)	78 (74)	81 (83)
ORF3 (HSP70h)	77 (83)	78 (84)	79 (85)	79 (86)	80 (87)	80 (86)	80 (87)	76 (81)
ORF4 (p61)	74 (79)	74 (79)	76 (80)	76 (79)	75 (79)	76 (79)	75 (79)	73 (74)
ORF5 (CP)	74 (76)	74 (78)	73 (75)	77 (78)	77 (79)	77 (78)	78 (78)	71 (72)
ORF6 (CPm)	72 (70)	73 (71)	73 (72)	73 (72)	73 (71)	73 (71)	73 (70)	70 (68)
ORF7 (p21)	81 (90)	80 (90)	82 (93)	84 (94)	84 (92)	84 (92)	85 (92)	77 (85)
ORF8 (p27)	74 (75)	79 (82)	81 (85)	80 (85)	79 (82)	79 (81)	79 (82)	75 (71)

The following isolates sequences, obtained from GenBank, were used ITMAR (EU715989), UW2 (Y10237), V2356 (JX669615), YD (KR080325), JERTE (KX192366), PONFERRADA (KX192367), TAIAN (KR736335).

## Supplementary Materials

The following are available online at http://www.mdpi.com/1999-4915/10/7/385/s1, RT-PCR/RFLPs based analysis in samples C118 and P8. Figure S1: Maximum likelihood phylogenetic trees inferred from the partial (a) RdRp, (b) HSP70h and (c) CP nucleotide sequences, Table S1: Primers used to complete the full-length genomic sequence of the Kyoto-2 and G15 3 isolates, Table S2: Identity scores in nt and aminoacids among all full genome LChV1 isolates.
