# Supplementary material for: High-Throughput Sequencing Reveals Further Diversity of Little Cherry Virus 1 with Implications for Diagnostics
Source: Viruses. 2018 Jul 21;10(7):385. doi: 10.3390/v10070385 (PMC6070981; doi:10.3390/v10070385)
Supplement: Supplementary file 1 [file viruses-10-00385-s001.zip › Supl M & M, Figures and tables/Fig S1.docx]

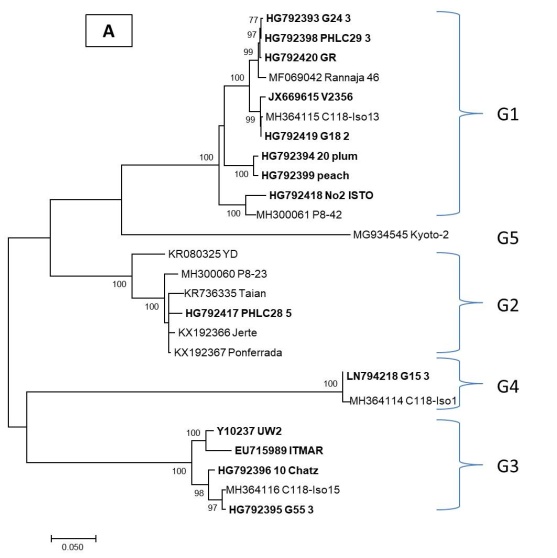

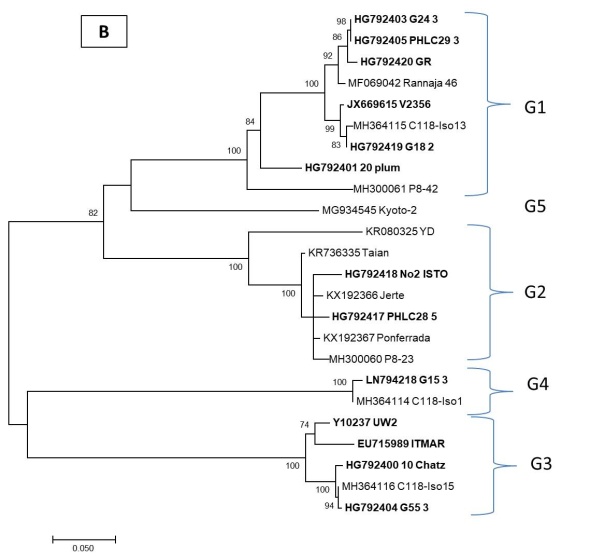


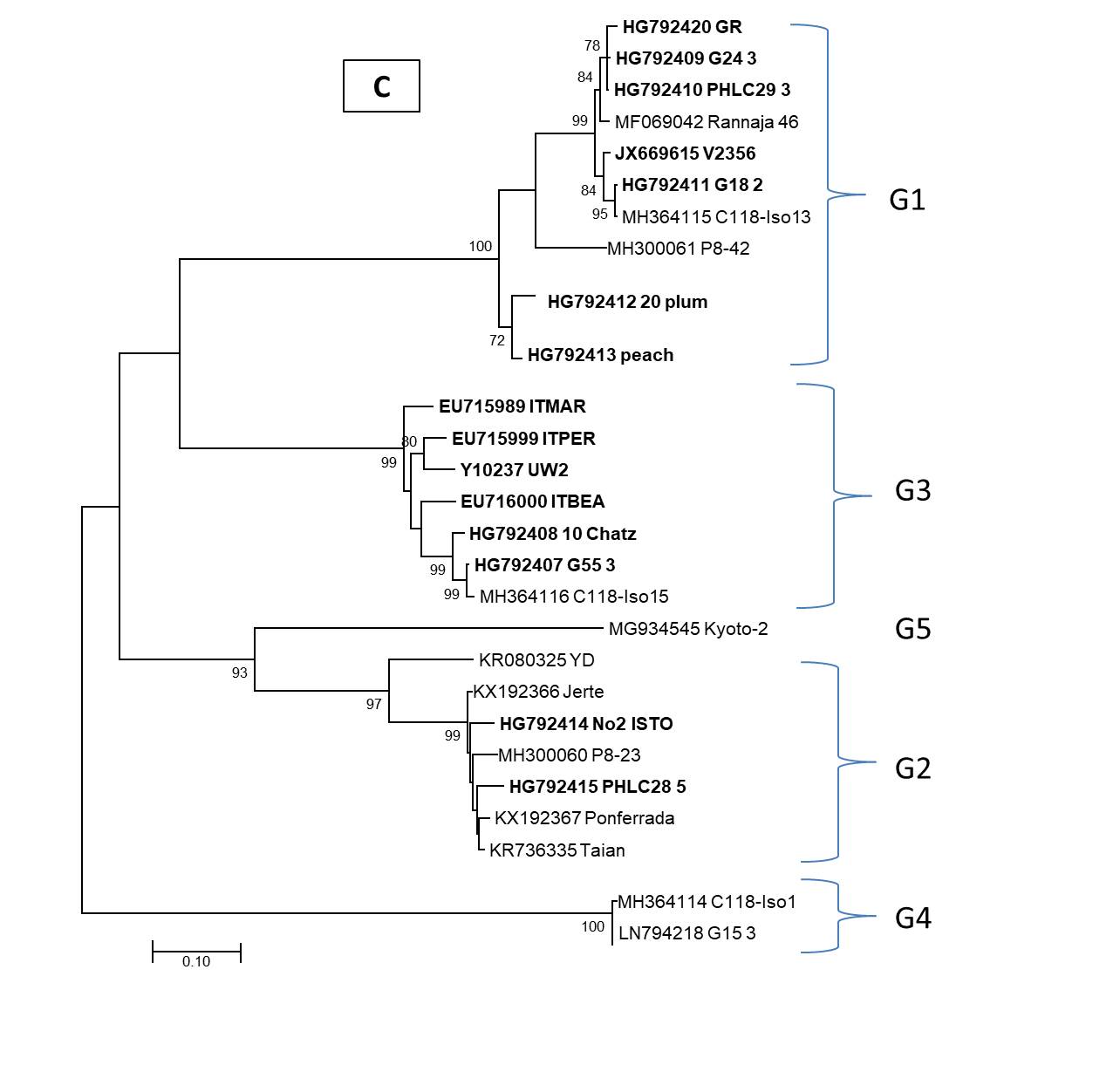


**Figure S1.** Maximum likelihood phylogenetic trees inferred from the partial a) RdRp, b) HSP70h and c) CP nucleotide sequences. The isolates included in a previous analysis (Katsiani et al., 2015) are shown in bold. All isolates are reported with their names followed by their accession numbers. All trees are midpoint-rooted. The numbers above or below each branch are the nonparametric bootstrap (NPB) values given as percentages of 500 replicates. Only NPB values with P > 70 are shown.
