# Supplementary material for: High-Throughput Sequencing Reveals Further Diversity of Little Cherry Virus 1 with Implications for Diagnostics
Source: Viruses. 2018 Jul 21;10(7):385. doi: 10.3390/v10070385 (PMC6070981; doi:10.3390/v10070385)

**RT-PCR/RFLPs based analysis in samples C118 and P8.**

To confirm the mixed infection with different LChV1 genotypes in C118 sweet cherry sample, the RT-PCR/restriction fragment length polymorphism (RFLP) approach was employed. Part of the viral CP region (559 bps) was amplified with RT-PCR using the degenerate primers CPiso up and CPiso do (Katsiani et al., 2015). Comparisons *in silico* with C118-Iso1, C118-Iso13 and C118-Iso15 have shown zero mismatches with the primers thus allowing their simultaneous amplification. At the same time, the sequences were imported into NEBcutter V2.0 (<http://nc2.neb.com/NEBcutter2/>) to obtain a list of enzymes that cut the particular fragment in a single site. Two of these were chosen, XhoI for C118-Iso13 (502 bps and 57 bps) and EcoRI for C118-Iso15 (297 bps and 262 bps). On the other hand, the recognition sites of XhoI and EcoRI were not present in C118-Iso1 sequence resulting in a single band after digestion (559 bps). The RT-PCR amplicon was purified using the NucleoTrap® extraction II kit (Macherey-Nagel, Düren, Germany), subsequently ligated into the pCR®4-TOPO® vector (Invitrogen-Life Technologies, Groningen, The Netherlands) and cloned into E. coli. Several colonies were selected and the recombinant plasmids were purified using the NucleoSpin® Plasmid kit (MachereyNagel, Düren, Germany). The purified plasmids were used as template in new RT-PCRs with the same primer mix. Then, 10 μl of several PCR amplicons were digested with 5 U of each endonuclease in the same tube under conditions recommended by the manufacturer (New England Biolabs). Lastly, the restriction fragments were electrophoresed in 2.5% agarose gels in TAE buffer, stained with ethidium bromide and visualised under UV light. The RFLP analysis has shown all three patterns representing the three isolates and was also confirmed with Sanger sequencing.


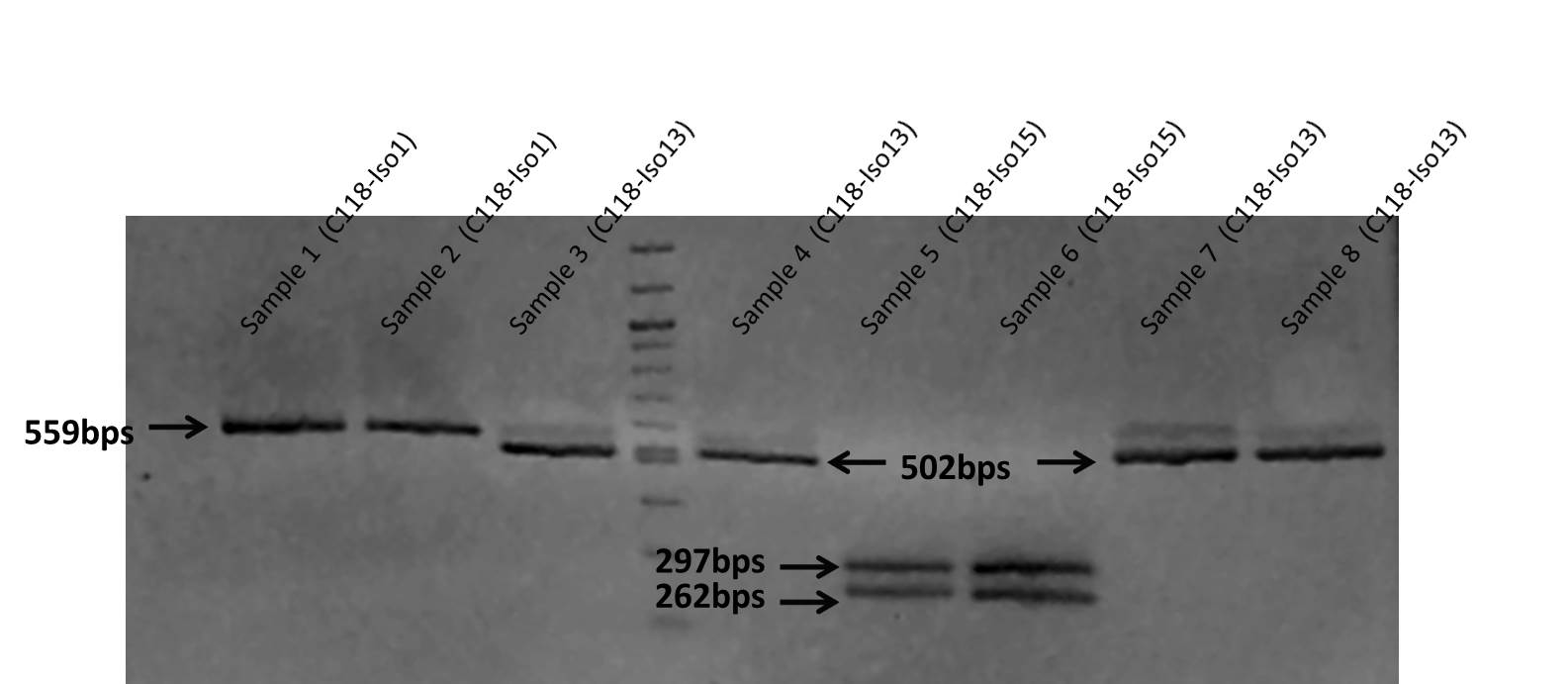


In order to confirm the mixed infection with different LChV1 genotypes in P8 sweet cherry sample, the RT-PCR/restriction fragment length polymorphism (RFLP) approach was employed. Part of the viral CP region (559 bps) was amplified with RT-PCR using the degenerate primers LChV1-P8-42-23-F (5’-CCRATYGTKMARCCTATWTGG-3’) and LChV1-P8-42-23-R (5’-GYGAAAAATCRAGYTTATCAAAATTYA-3’). Comparisons *in silico* with P8-42 and P8-23 showed zero mismatches with the primers, thus allowing their simultaneous amplification. At the same time, the sequences were analyzed by Geneious software to obtain a list of enzymes that cut in a single site the particular fragment. Two of these were chosen, EcoRV for P8-23 (417 bps and 142 bps), which recognition site was not present in P8-42, and BamHI for P8-42 (484 bps and 75 bps), which recognition site was not present in P8-23. The RT-PCR amplicon was purified using the mi-PCR Purification kit (Μetabion international AG, Martinsried, Germany), subsequently ligated into the pGEM-T Easy vector (Promega Corporation, Madison, USA) and cloned into E. coli. Several colonies were selected and the recombinant plasmids were purified using the (PureYield^TM^ Plasmid Miniprep Sysytem, Promega Corporation, Madison, USA). The purified plasmids were used as template in new RT-PCRs with the same primer mix. Then, 10 μl of several PCR amplicons were digested with 5 U of each endonuclease in the same tube under conditions recommended by the manufacturer (New England Biolabs). Lastly, the restriction fragments were electrophoresed in 2.5% agarose gels in TAE buffer, stained with ethidium bromide and visualised under UV light. The RFLP analysis has shown all two patterns representing the two isolates and was also confirmed with Sanger sequencing.


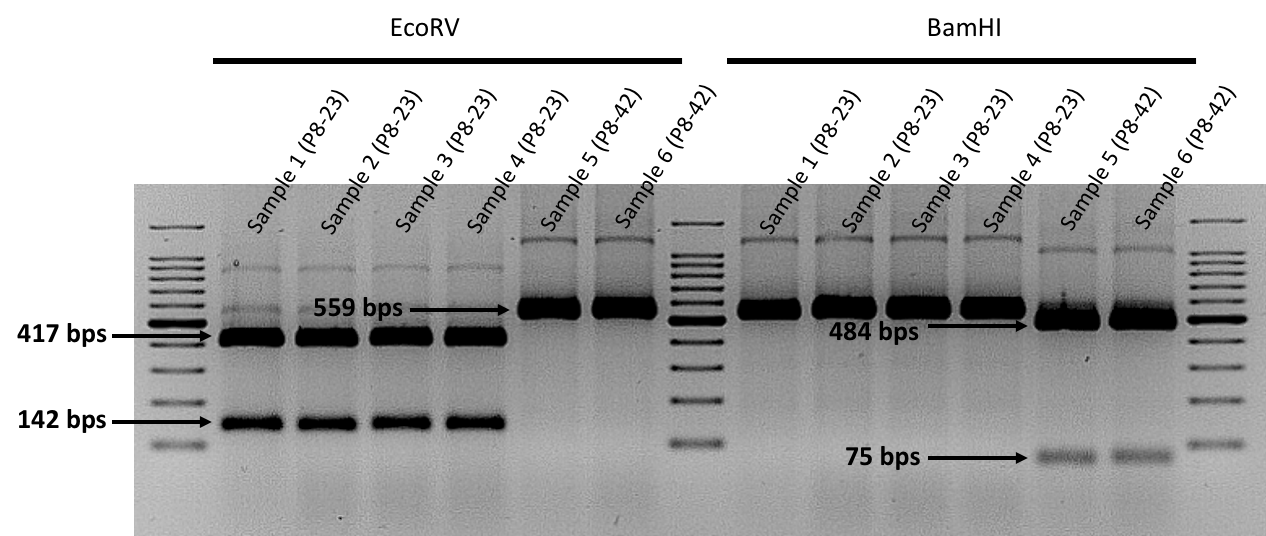

Supplement: Supplementary file 1 [file viruses-10-00385-s001.zip › Supl M & M, Figures and tables/Supplementary Material 21.7.2018.docx]
