# Supplementary material for: High-Throughput Sequencing Reveals Further Diversity of Little Cherry Virus 1 with Implications for Diagnostics
Source: Viruses. 2018 Jul 21;10(7):385. doi: 10.3390/v10070385 (PMC6070981; doi:10.3390/v10070385)
Supplement: Supplementary file 1 [file viruses-10-00385-s001.zip › Supl M & M, Figures and tables/Table S1.docx]

**Table S1:** Primers used to complete the full-length genomic sequence of the Kyoto-2 isolate and G15 3. The position of the first 5΄ nucleotide of each primer is according to the Kyoto-2 and G15 3 sequence, respectively.

| **Kyoto-2** | **Primer pair** | **Primer sequence (5’-> 3’)** | **5’ position** | **Tm** |  |
| --- | --- | --- | --- | --- | --- |
|  | LChV1-likeRace5 | GGGTGCAGAACGAGCGTCAGCTAATGC | 237 | 70°C |  |
|  | LChV1-likeF1  LChV1-likeR1 | ATTGAGCTTGCTTCTAAGGC  GGTAGTAACATAATGAAGTCC | 1435  2577 | 56°C |  |
|  |  |  |  |  |  |
|  | LChV1-likeF2  LChV1-likeR2 | TGATGTCGTCGTATCTGATG CATAATATAGAGGACCTTCGC | 3972  4418 | 56°C |  |
|  | LChV1-likeF3  LChV1-likeR3 | TCTTCGTCTTACAATGTAGGG  CCTGAATCATATTTATCGAGG | 5509  5798 | 56°C |  |
|  |  |  |  |  |  |
|  | LChV1-likeF4  LChV1-likeR4 | AAGTGTTTACGCAGTCAACG  TCACTCAGCTTGAAATTGTCG | 7045  7902 | 56°C |  |
|  |  |  |  |  |  |
|  | LChV1-likeF5  LChV1-likeR5 | AATTGAGAATAAGTGTCTCG  GGTACAAATGGAGATTTATC | 8095  8708 | 52°C |  |
|  |  |  |  |  |  |
|  | LChV1-likeF6  LChV1-likeR6 | CTCATGGTTTATTAGATGTAGG  TTCGTTGACTTCAAATAATTGC | 9719  11376 | 56°C |  |
|  |  |  |  |  |  |
|  | LChV1-likeF7  LChV1-likeR7 | TGCACAATCTTTGAGTGACC  CATTAGTGTAGCTTATGTCTA | 11430  12353 | 54°C |  |
|  | LChV1-likeF8  LChV1-likeR8 | GTAGTTGGTATTTGAACGATGG  GATATCTCACGAAATCAGTCAA | 13808  14218 | 58°C |  |
|  | LChV1-likeF9  LChV1-likeR9 | TGTAGTCGCAGCTCTTACTG  CTCACTTATATCTCTCTCGTC | 15369  15684 | 58°C |  |
|  | LChV1-likeRace3 | GTAACTTCGAGCATGACGCTGATGGAC | 16295 | 70°C |  |
| **G15 3** | 214 F  777 R | CATTGGCTGACGCTCGTT  GCAACTCAGGGAAGTTTTTGTCA | 214  777 | 62^ο^C |  |
|  | 701 F  1.301 R | GATTTATGTCCCCTCTGCTGT  AGCATATGATTTTGTTTCACTGCT | 701  1.301 | 61°C |  |
|  | 1.182 F  1.734 R | ACTGAGTTCGCTGTAGCTCT  ACCAACAGAACCCATTGTCA | 1.182  1.734 | 61°C |  |
|  | 1.676 F  2.359 R | TTACTGCAGGACACCGTCTG  TCTGCCACAGGATTACAAATGT | 1.676  2.359 | 62°C |  |
|  | 2.264 F  2.915 R | GCATGAAGGTCGGCATTTTGT  CCCATTGATGTTTCTCATTGGGA | 2.264  2.915 | 62°C |  |
|  | 2.808 F  3.253 R | ACCAAATTAGAAACTTTCCCGAG  TGCAAGAACAATTGGCGGAT | 2.808  3.253 | 60°C |  |
|  | 3.140 F  3.658 R | GGGGATTCGACTGGCCGA  TGACGACGCAACCAGTCAAA | 3.140  3.658 | 64°C |  |
|  | 3.500 F  4.386 R | TGAGTTATCTGATGCGGTTGA  CAAAAGAATCTCGCAACACGT | 3.500  4.386 | 61°C |  |
|  | 4.321 F  5.031 R | TGTTTAAGCATTACATTATAGCCAAGT  AAACAGCGACACCGGCTG | 4.321  5.031 | 62°C |  |
|  | 4.941 F  5.807 R | CATGTCGCGACGCATGTTG  CCTCATTCCTGAGTCATATTTGTC | 4.941  5.807 | 61°C |  |
|  | 5.731 F  6.523 R | AGATTGAAAGCTTGACCTTATTACTT  TCTGCAGGACAACGATAACTT | 5.731  6.523 | 61°C |  |
|  | 6.408 F  7.100 R | GAGAACCAGATTCCTTACTCTGT  GAAGACAACCCGTTGAAGGT | 6.408  7.100 | 61°C |  |
|  | 7.024 F  7.747 R | TTTCCTGCCCGACCTAGTTT  TTTCAATAAGATCACCCATTTCATCA | 7.024  7.747 | 62°C |  |
|  | 7.665 F  8.390 R | TGGTAGAATAAAGTATTGTCTTGGTG TTATACTTCATACTCACGAACCTTTC | 7.665  8.390 | 61°C |  |
|  | 8.316 F  9.180 R | TCGTGATCTCATGGGAGCAT  CCACAAAGTCATTCTCCTTAGGA | 8.316  9.180 | 62°C |  |
|  | 9.120 F  9.943 R | GAACCCTCTGCAGCTGCTA  ATATACCCAGAACCTAACTCAGAC | 9.120  9.943 | 61°C |  |
|  | 9.861 F  10.445 R | AGTTTAAACTTCCTCGAAAATGACT ACTCACTATCTCCATTCCTCTTTAA | 9.861  10.445 | 61°C |  |
|  | 10.266 F  11.106 R | TGAGGAACGCTTAGTAGTTCG  GCATGCTTTGAGTGTAAAGAGGT | 10.266  11.106 | 61°C |  |
|  | 10.994 F  11.749 R | GGAGAGTTATACTGAACGATCTGA  GCTCTCTTTGAACAAAACAACCT | 10.994  11.749 | 61°C |  |
|  | 11.683 F  12.544 R | GAAGGAATGAACTAGCATTTAGAGAT GAGCCTTAGGTAATTTTTCGATGT | 11.683  12.544 | 61°C |  |
|  | 12.474 F  13.165 R | AAAATCAACATTGACTCAAGATGAATT  GTTCATCATAAGTCATTGATGCTTTG | 12.474  13.165 | 61°C |  |
|  | 13.090 F  13.844 R | GGAGCTGGTCCGGGTAAG  TTTGTGATTCGGTCCGTCGT | 13.090  13.844 | 63°C |  |
|  | 13.760 F  14.513 R | ATCACCTAGGTATAGCATCGAAAT  ATGGTGGTTAGTCATATCAGAACT | 13.760  14.513 | 61°C |  |
|  | 14.276 F  15.150 R | CGACAGATTTCATAGTTATGTTTTCAC GCGAATTGTTTATCAATACCAAGCT | 14.276  15.150 | 61°C |  |
|  | 15.072 F  15.830 R | TGCTAGAATTGCTTAGGATTGGT TGTAAAACCTTTCAAACATTCAGATTT | 15.072  15.830 | 61°C |  |
|  | 15.718 F  16.417 R | AGTTAAAGATGTTATCGGAGGAGT CGGGATTGAATTAAATATTTCAAACGG | 15.718  16.417 | 61°C |  |
|  | 16.151 F  16.904 R | CAGGAACACCTTTGATAAGATGTT  CCTAGGTGGTCTATCCTATCGGA | 16.151  16.904 | 61°C |  |
