# Supplementary material for: High-Throughput Sequencing Reveals Further Diversity of Little Cherry Virus 1 with Implications for Diagnostics
Source: Viruses. 2018 Jul 21;10(7):385. doi: 10.3390/v10070385 (PMC6070981; doi:10.3390/v10070385)
Supplement: Supplementary file 1 [file viruses-10-00385-s001.zip › Supl M & M, Figures and tables/table S2.docx]

**Table S2.** Identity scores in nt (gray color lower half) and aminoacids (black color upper half) among all full genome LChV1 isolates.

| Gene  (nt \ aa) | Isolates | G15 3 | YD | Jerte | V2356 | Ponferrada | Taian | UW2 | ITMAR | Kyoto-2 | C118-Iso1 | C118-Iso13 | C118-Iso15 | P8-23 | P8-42 |
| --- | --- | --- | --- | --- | --- | --- | --- | --- | --- | --- | --- | --- | --- | --- | --- |
| ORF1a | G15 3 |  | 76 | 77 | 76 | 76 | 76 | 76 | 75 | 76 | 99 | 77 | 75 | 76 | 77 |
|  | YD | 72 |  | 91 | 82 | 90 | 90 | 80 | 79 | 83 | 76 | 82 | 79 | 90 | 82 |
|  | Jerte | 72 | 89 |  | 83 | 97 | 97 | 80 | 79 | 83 | 77 | 83 | 80 | 97 | 83 |
|  | V2356 | 72 | 76 | 76 |  | 82 | 82 | 81 | 80 | 83 | 76 | 98 | 81 | 82 | 91 |
|  | Ponferrada | 71 | 89 | 97 | 76 |  | 97 | 80 | 78 | 82 | 76 | 82 | 79 | 96 | 82 |
|  | Taian | 71 | 88 | 97 | 76 | 97 |  | 80 | 79 | 82 | 76 | 82 | 79 | 96 | 82 |
|  | UW2 | 70 | 74 | 74 | 75 | 74 | 74 |  | 94 | 80 | 76 | 81 | 93 | 80 | 81 |
|  | ITMAR | 70 | 73 | 73 | 74 | 73 | 73 | 94 |  | 79 | 75 | 81 | 92 | 79 | 80 |
|  | Kyoto-2 | 71 | 75 | 75 | 76 | 75 | 75 | 74 | 74 |  | 76 | 83 | 80 | 82 | 83 |
|  | C118-Iso1 | 99 | 72 | 72 | 72 | 71 | 71 | 70 | 70 | 71 |  | 77 | 75 | 77 | 77 |
|  | C118-Iso13 | 72 | 76 | 76 | 98 | 76 | 76 | 75 | 75 | 76 | 72 |  | 81 | 82 | 91 |
|  | C18-Iso15 | 71 | 74 | 74 | 75 | 74 | 74 | 93 | 92 | 74 | 71 | 75 |  | 80 | 81 |
|  | P8-23 | 72 | 76 | 76 | 89 | 76 | 76 | 74 | 74 | 76 | 71 | 89 | 74 |  | 82 |
|  | P8-42 | 72 | 89 | 96 | 76 | 96 | 96 | 74 | 73 | 75 | 72 | 76 | 74 | 76 |  |
| ORF1b | G15 3 |  | 92 | 91 | 92 | 92 | 90 | 92 | 88 | 92 | 99 | 92 | 93 | 92 | 92 |
|  | YD | 80 |  | 96 | 94 | 96 | 96 | 96 | 93 | 94 | 91 | 94 | 95 | 97 | 95 |
|  | Jerte | 80 | 92 |  | 94 | 98 | 98 | 93 | 92 | 93 | 90 | 94 | 94 | 98 | 95 |
|  | V2356 | 81 | 83 | 84 |  | 94 | 93 | 94 | 90 | 94 | 92 | 100 | 94 | 94 | 98 |
|  | Ponferrada | 81 | 92 | 98 | 84 |  | 98 | 94 | 92 | 93 | 91 | 94 | 94 | 98 | 94 |
|  | Taian | 80 | 92 | 97 | 84 | 98 |  | 94 | 92 | 93 | 90 | 93 | 94 | 94 | 94 |
|  | UW2 | 80 | 83 | 82 | 82 | 82 | 82 |  | 97 | 93 | 92 | 94 | 98 | 90 | 90 |
|  | ITMAR | 80 | 82 | 80 | 81 | 80 | 80 | 96 |  | 89 | 88 | 90 | 93 | 94 | 95 |
|  | Kyoto-2 | 80 | 80 | 81 | 83 | 81 | 81 | 80 | 79 |  | 92 | 94 | 94 | 91 | 91 |
|  | C118-Iso1 | 99 | 80 | 80 | 81 | 80 | 80 | 80 | 80 | 80 |  | 92 | 92 | 94 | 98 |
|  | C118-Iso13 | 81 | 83 | 84 | 99 | 84 | 84 | 82 | 81 | 83 | 81 |  | 94 | 95 | 94 |
|  | C118-Iso15 | 81 | 83 | 81 | 82 | 82 | 81 | 94 | 94 | 80 | 80 | 82 |  | 95 | 94 |
|  | P8-23 | 81 | 92 | 98 | 93 | 98 | 97 | 82 | 80 | 81 | 80 | 84 | 82 |  | 95 |
|  | P8-42 | 80 | 83 | 84 | 84 | 84 | 83 | 82 | 81 | 84 | 81 | 93 | 82 | 84 |  |
| ORF2 | G15 3 |  | 70 | 70 | 77 | 67 | 74 | 67 | 67 | 83 | 100 | 77 | 74 | 70 | 70 |
|  | YD | 76 |  | 96 | 70 | 90 | 87 | 77 | 74 | 70 | 70 | 70 | 77 | 96 | 74 |
|  | Jerte | 74 | 96 |  | 70 | 93 | 90 | 77 | 74 | 70 | 70 | 70 | 77 | 100 | 74 |
|  | V2356 | 76 | 79 | 78 |  | 70 | 70 | 70 | 64 | 80 | 77 | 100 | 77 | 70 | 87 |
|  | Ponferrada | 70 | 92 | 95 | 78 |  | 90 | 74 | 67 | 74 | 67 | 70 | 70 | 93 | 67 |
|  | Taian | 73 | 91 | 94 | 78 | 96 |  | 70 | 64 | 74 | 74 | 70 | 70 | 90 | 70 |
|  | UW2 | 70 | 80 | 78 | 78 | 78 | 77 |  | 80 | 77 | 67 | 70 | 87 | 77 | 74 |
|  | ITMAR | 72 | 79 | 77 | 74 | 77 | 76 | 91 |  | 70 | 67 | 64 | 83 | 74 | 64 |
|  | Kyoto-2 | 81 | 81 | 79 | 81 | 78 | 78 | 82 | 79 |  | 83 | 80 | 83 | 74 | 70 |
|  | C118-Iso1 | 100 | 77 | 74 | 77 | 72 | 73 | 71 | 72 | 81 |  | 77 | 74 | 70 | 70 |
|  | C118-Iso13 | 77 | 81 | 79 | 98 | 79 | 79 | 78 | 75 | 81 | 77 |  | 77 | 70 | 87 |
|  | C118-Iso15 | 74 | 81 | 78 | 79 | 78 | 78 | 92 | 92 | 81 | 74 | 79 |  | 77 | 77 |
|  | P8-23 | 74 | 95 | 99 | 80 | 95 | 94 | 77 | 76 | 79 | 73 | 80 | 77 |  | 74 |
|  | P8-42 | 73 | 81 | 79 | 91 | 77 | 79 | 78 | 75 | 81 | 74 | 92 | 79 | 80 |  |
| ORF3 | G15 3 |  | 83 | 83 | 81 | 83 | 83 | 81 | 79 | 81 | 99 | 80 | 82 | 82 | 80 |
|  | YD | 76 |  | 96 | 85 | 95 | 95 | 85 | 84 | 86 | 82 | 84 | 85 | 94 | 84 |
|  | Jerte | 76 | 92 |  | 86 | 99 | 99 | 85 | 85 | 87 | 82 | 85 | 86 | 98 | 85 |
|  | V2356 | 74 | 79 | 80 |  | 85 | 86 | 82 | 82 | 85 | 80 | 99 | 83 | 85 | 93 |
|  | Ponferrada | 76 | 91 | 98 | 79 |  | 98 | 85 | 84 | 86 | 82 | 85 | 86 | 98 | 84 |
|  | Taian | 76 | 91 | 98 | 80 | 98 |  | 86 | 85 | 87 | 83 | 86 | 86 | 97 | 85 |
|  | UW2 | 76 | 79 | 78 | 77 | 79 | 79 |  | 96 | 84 | 81 | 82 | 96 | 85 | 83 |
|  | ITMAR | 75 | 78 | 78 | 76 | 78 | 78 | 96 |  | 83 | 79 | 81 | 94 | 84 | 82 |
|  | Kyoto-2 | 76 | 79 | 80 | 79 | 80 | 80 | 78 | 77 |  | 81 | 85 | 85 | 86 | 85 |
|  | C118-Iso1 | 99 | 76 | 76 | 75 | 76 | 76 | 76 | 75 | 76 |  | 80 | 82 | 82 | 80 |
|  | C118-Iso13 | 74 | 79 | 79 | 98 | 79 | 79 | 76 | 76 | 79 | 74 |  | 83 | 85 | 93 |
|  | C118-Iso15 | 76 | 78 | 79 | 77 | 79 | 79 | 93 | 93 | 78 | 76 | 77 |  | 86 | 83 |
|  | P8-23 | 76 | 91 | 98 | 80 | 98 | 97 | 79 | 78 | 80 | 76 | 79 | 79 |  | 84 |
|  | P8-42 | 75 | 79 | 79 | 90 | 79 | 79 | 77 | 77 | 79 | 74 | 90 | 77 | 79 |  |
| ORF4 | G15 3 |  | 75 | 74 | 76 | 74 | 74 | 75 | 75 | 74 | 98 | 76 | 75 | 74 | 77 |
|  | YD | 72 |  | 89 | 79 | 90 | 90 | 80 | 78 | 79 | 75 | 79 | 79 | 90 | 81 |
|  | Jerte | 73 | 90 |  | 79 | 99 | 97 | 80 | 79 | 79 | 75 | 79 | 79 | 97 | 80 |
|  | V2356 | 74 | 76 | 76 |  | 79 | 79 | 79 | 79 | 80 | 75 | 98 | 78 | 79 | 91 |
|  | Ponferrada | 73 | 90 | 98 | 76 |  | 98 | 80 | 79 | 79 | 75 | 79 | 79 | 98 | 80 |
|  | Taian | 73 | 89 | 97 | 76 | 98 |  | 80 | 80 | 79 | 75 | 79 | 79 | 96 | 80 |
|  | UW2 | 72 | 74 | 75 | 75 | 75 | 75 |  | 94 | 79 | 76 | 79 | 94 | 80 | 80 |
|  | ITMAR | 72 | 74 | 75 | 75 | 75 | 75 | 95 |  | 79 | 75 | 79 | 93 | 79 | 80 |
|  | Kyoto-2 | 73 | 76 | 75 | 76 | 76 | 75 | 74 | 74 |  | 74 | 80 | 79 | 78 | 81 |
|  | C118-Iso1 | 99 | 73 | 73 | 74 | 74 | 73 | 72 | 72 | 73 |  | 75 | 75 | 75 | 77 |
|  | C118-Iso13 | 74 | 76 | 75 | 98 | 76 | 75 | 75 | 75 | 76 | 73 |  | 79 | 79 | 91 |
|  | C118-Iso15 | 72 | 74 | 75 | 75 | 75 | 75 | 93 | 93 | 76 | 72 | 75 |  | 79 | 79 |
|  | P8-23 | 73 | 89 | 97 | 76 | 98 | 97 | 75 | 75 | 76 | 73 | 76 | 75 |  | 80 |
|  | P8-42 | 73 | 77 | 76 | 91 | 77 | 76 | 76 | 75 | 76 | 73 | 90 | 75 | 77 |  |
| ORF5 | G15 3 |  | 73 | 74 | 72 | 74 | 74 | 72 | 73 | 72 | 99 | 73 | 72 | 73 | 72 |
|  | YD | 72 |  | *90* | 79 | 89 | 89 | 79 | 78 | 78 | 73 | 79 | 79 | 88 | 78 |
|  | Jerte | 72 | 89 |  | 78 | 98 | 98 | 81 | 79 | 79 | 74 | 78 | 79 | 96 | 77 |
|  | V2356 | 70 | 76 | 75 |  | 79 | 78 | 77 | 76 | 75 | 72 | 98 | 75 | 77 | 89 |
|  | Ponferrada | 72 | 89 | 98 | 75 |  | 99 | 80 | 78 | 78 | 73 | 78 | 79 | 96 | 77 |
|  | Taian | 72 | 89 | 98 | 75 | 98 |  | 80 | 78 | 78 | 74 | 78 | 79 | 96 | 77 |
|  | UW2 | 72 | 75 | 76 | 75 | 76 | 75 |  | 92 | 78 | 72 | 76 | 90 | 79 | 75 |
|  | ITMAR | 72 | 74 | 75 | 75 | 75 | 75 | 94 |  | 76 | 72 | 76 | 90 | 78 | 75 |
|  | Kyoto-2 | 71 | 77 | 77 | 73 | 77 | 78 | 74 | 74 |  | 71 | 75 | 76 | 77 | 75 |
|  | C118-Iso1 | 99 | 72 | 72 | 70 | 72 | 72 | 72 | 72 | 71 |  | 73 | 72 | 74 | 72 |
|  | C118-Iso13 | 71 | 77 | 76 | 98 | 75 | 76 | 75 | 75 | 73 | 70 |  | 75 | 77 | 90 |
|  | C118-Iso15 | 71 | 75 | 75 | 74 | 75 | 75 | 92 | 92 | 73 | 71 | 74 |  | 78 | 75 |
|  | P8-23 | 72 | 88 | 97 | 75 | 97 | 75 | 97 | 97 | 75 | 75 | 77 | 72 |  | 77 |
|  | P8-42 | 70 | 76 | 75 | 91 | 75 | 74 | 74 | 74 | 72 | 70 | 91 | 74 | 74 |  |
| ORF6 | G15 3 |  | 68 | 67 | 65 | 67 | 67 | 68 | 66 | 68 | 99 | 65 | 66 | 67 | 66 |
|  | YD | 69 |  | 88 | 72 | 88 | 88 | 70 | 71 | 72 | 67 | 72 | 71 | 88 | 72 |
|  | Jerte | 69 | 90 |  | 71 | 97 | 97 | 70 | 71 | 71 | 66 | 71 | 71 | 96 | 71 |
|  | V2356 | 68 | 74 | 73 |  | 70 | 70 | 69 | 69 | 72 | 64 | 96 | 70 | 71 | 84 |
|  | Ponferrada | 69 | 90 | 98 | 73 |  | 98 | 69 | 71 | 71 | 66 | 71 | 70 | 97 | 70 |
|  | Taian | 70 | 90 | 97 | 73 | 98 |  | 69 | 71 | 70 | 67 | 71 | 70 | 96 | 71 |
|  | UW2 | 70 | 73 | 72 | 72 | 72 | 72 |  | 90 | 71 | 67 | 69 | 91 | 69 | 67 |
|  | ITMAR | 69 | 74 | 73 | 72 | 73 | 74 | 92 |  | 70 | 66 | 69 | 88 | 71 | 67 |
|  | Kyoto-2 | 70 | 73 | 73 | 73 | 73 | 73 | 73 | 72 |  | 68 | 72 | 71 | 71 | 70 |
|  | C118-Iso1 | 99 | 69 | 69 | 68 | 69 | 70 | 70 | 69 | 70 |  | 65 | 66 | 67 | 65 |
|  | C118-Iso13 | 68 | 74 | 74 | 97 | 73 | 73 | 72 | 72 | 73 | 68 |  | 71 | 71 | 84 |
|  | C118-Iso15 | 69 | 73 | 72 | 73 | 73 | 73 | 93 | 90 | 73 | 69 | 73 |  | 71 | 68 |
|  | P8-23 | 70 | 89 | 97 | 74 | 97 | 97 | 73 | 74 | 73 | 70 | 74 | 73 |  | 71 |
|  | P8-42 | 69 | 74 | 74 | 87 | 74 | 74 | 72 | 71 | 71 | 69 | 87 | 72 | 74 |  |
| ORF7 | G15 3 |  | 87 | 88 | 85 | 88 | 88 | 87 | 88 | 85 | 100 | 85 | 87 | 88 | 85 |
|  | YD | 78 |  | 97 | 95 | 97 | 97 | 91 | 93 | 94 | 87 | 95 | 90 | 97 | 95 |
|  | Jerte | 78 | 92 |  | 93 | 100 | 100 | 91 | 92 | 92 | 88 | 93 | 90 | 100 | 92 |
|  | V2356 | 76 | 81 | 82 |  | 93 | 93 | 88 | 88 | 93 | 85 | 100 | 87 | 93 | 96 |
|  | Ponferrada | 78 | 93 | 99 | 82 |  | 100 | 91 | 92 | 92 | 88 | 93 | 90 | 100 | 92 |
|  | Taian | 78 | 91 | 98 | 82 | 98 |  | 91 | 92 | 92 | 88 | 93 | 90 | 100 | 92 |
|  | UW2 | 76 | 83 | 82 | 81 | 83 | 82 |  | 94 | 90 | 87 | 88 | 98 | 91 | 90 |
|  | ITMAR | 76 | 86 | 87 | 81 | 88 | 87 | 91 |  | 90 | 88 | 88 | 93 | 92 | 90 |
|  | Kyoto-2 | 77 | 84 | 84 | 82 | 84 | 85 | 80 | 81 |  | 85 | 93 | 88 | 92 | 93 |
|  | C118-Iso1 | 99 | 78 | 78 | 76 | 78 | 78 | 76 | 76 | 77 |  | 85 | 87 | 88 | 85 |
|  | C118-Iso13 | 75 | 81 | 81 | 99 | 81 | 81 | 81 | 81 | 82 | 75 |  | 87 | 93 | 96 |
|  | C118-Iso15 | 77 | 84 | 82 | 81 | 83 | 82 | 95 | 89 | 80 | 77 | 81 |  | 90 | 89 |
|  | P8-23 | 78 | 91 | 97 | 81 | 97 | 97 | 81 | 86 | 84 | 78 | 81 | 82 |  | 92 |
|  | P8-42 | 76 | 82 | 83 | 93 | 82 | 82 | 82 | 83 | 82 | 76 | 93 | 82 | 82 |  |
| ORF8 | G15 3 |  | 68 | 72 | 72 | 73 | 73 | 72 | 67 | 69 | 97 | 72 | 72 | 73 | 67 |
|  | YD | 73 |  | 85 | 87 | 84 | 84 | 83 | 77 | 86 | 67 | 86 | 82 | 84 | 84 |
|  | Jerte | 73 | 89 |  | 90 | 98 | 99 | 82 | 77 | 82 | 71 | 89 | 81 | 99 | 84 |
|  | V2356 | 74 | 81 | 83 |  | 90 | 90 | 82 | 78 | 85 | 71 | 98 | 83 | 90 | 88 |
|  | Ponferrada | 73 | 89 | 98 | 84 |  | 99 | 81 | 77 | 81 | 72 | 89 | 81 | 99 | 84 |
|  | Taian | 72 | 88 | 98 | 84 | 98 |  | 81 | 76 | 81 | 72 | 89 | 81 | 100 | 84 |
|  | UW2 | 73 | 79 | 79 | 79 | 78 | 79 |  | 89 | 82 | 70 | 81 | 92 | 81 | 79 |
|  | ITMAR | 71 | 77 | 79 | 77 | 78 | 77 | 92 |  | 75 | 65 | 77 | 85 | 76 | 72 |
|  | Kyoto-2 | 75 | 80 | 79 | 81 | 79 | 79 | 79 | 74 |  | 68 | 84 | 81 | 81 | 83 |
|  | C118-Iso1 | 99 | 71 | 71 | 71 | 71 | 70 | 71 | 69 | 73 |  | 71 | 70 | 72 | 66 |
|  | C118-Iso13 | 72 | 80 | 81 | 98 | 82 | 81 | 76 | 74 | 81 | 71 |  | 82 | 89 | 87 |
|  | C118-Iso15 | 71 | 77 | 77 | 78 | 77 | 76 | 93 | 89 | 77 | 71 | 77 |  | 81 | 80 |
|  | P8-23 | 71 | 87 | 98 | 82 | 98 | 98 | 77 | 76 | 78 | 70 | 81 | 77 |  | 84 |
|  | P8-42 | 69 | 80 | 81 | 88 | 81 | 81 | 76 | 73 | 78 | 69 | 88 | 77 | 80 |  |
